# Zero average values of cosmological perturbations as an indispensable condition for the theory and simulations

**DOI:** 10.1140/epjc/s10052-015-3598-z

**Published:** 2015-08-22

**Authors:** Maxim Eingorn, Maxim Brilenkov, Branislav Vlahovic

**Affiliations:** CREST and NASA Research Centers, North Carolina Central University, Fayetteville st. 1801, Durham, NC 27707 USA; Department of Theoretical Physics and Astronomical Observatory, Odessa National University, Dvoryanskaya st. 2, Odessa, 65082 Ukraine

## Abstract

We point out a weak side of the commonly used determination of scalar cosmological perturbations lying in the fact that their average values can be nonzero for some matter distributions. It is shown that introduction of the finite-range gravitational potential instead of the infinite-range one resolves this problem. The concrete illustrative density profile is investigated in detail in this connection.

## Introduction

As is generally known, if a certain theory uses an average value $\bar{f}$ of a physical quantity *f* as its zeroth-order approximation ($f\approx \bar{f}$) and a deviation from this value $\delta f=f-\bar{f}$ as a quantity of the first order of smallness, then the average value of this deviation $\overline{\delta f}$ is equal to zero: $\overline{\delta f}=\overline{(f-\bar{f})}=0$. This clear argumentation is relevant, in particular, in the context of cosmological perturbations, if we assume that the homogeneous Friedmann–Lemaitre–Robertson–Walker (FLRW) geometry represents an average geometry for our cosmological spacetime. Indeed, proceeding on this assumption, the scale factor, which enters into the background FLRW metric and does not depend on the spatial location, actually describes the averaged metric coefficients originating from the averaged material sources. Here and in what follows we imply averaging over the volume in the comoving coordinates. Therefore, one immediately arrives at the inevitable conclusion that when the first-order perturbation theory is constructed against the homogeneous FLRW background, the spatial averaging procedure must give zero for an arising small correction to any unperturbed metric coefficient. The scale factor is otherwise determined incorrectly. Of course, the backreaction effect due to nonlinearity of general relativity leads to nonzero corrections to the FLRW background scale factor, but these corrections are of the second-order of smallness, so we do not take them into account. Actually, the discussed requirement of zero average values of first-order cosmological perturbations represents an indispensable condition for the corresponding theory and computer simulations based on the predicted equations of galaxy dynamics.

However, as we demonstrate explicitly in this paper, there exists a possibility of such matter distributions which lead to nonzero average values of the first-order metric corrections. Namely, in the framework of the mechanical approach to cosmological problems at the late stage of the Universe evolution we give a concrete example of a rest mass density profile for which the standard formula determining the scalar perturbations results in their nonzero average values. Since exactly this formula underlies the modern *N*-body simulations which play an extremely important role for the structure formation analysis, the discovered weak point must be eliminated in order to be fully confident in their predictions. We suggest avoiding this challenge without exceeding the limits of the conventional $\Lambda$CDM model, by cutting off the nonrelativistic gravitational potentials of cosmic bodies/inhomogeneities (e.g., galaxies).

The paper is structured in the following way. First, we enumerate briefly some basic achievements of the mechanical description of cosmological perturbations which may be associated with discrete cosmology in the nonrelativistic limit. Second, we prove that the commonly used infinite-range gravitational potential can be characterized by the nonzero average value, and then introduce the finite-range one in order to resolve this problem. Finally, we summarize laconically our results.

## Mechanical description of cosmological perturbations

According to the mechanical approach, developed in [1–3] in the framework of the conventional $\Lambda$CDM model (see also the related recent papers [4–7] where some similar issues and ideas are touched upon in the same spirit), the scalar cosmological perturbations in the late Universe with flat spatial topology can be described by the perturbed FLRW metric

2.1 $$\begin{aligned} \mathrm{d}s^2\approx a^2[(1+2\Phi )\mathrm{d}\eta ^2-(1-2\Phi )\delta _{\alpha \beta }\mathrm{d}x^{\alpha }\mathrm{d}x^{\beta }], \end{aligned}$$

where $a(\eta )$ is the scale factor,

2.2 $$\begin{aligned} \Phi (\eta ,\mathbf{r})=\frac{\varphi (\mathbf{r})}{c^2a(\eta )},\quad \triangle \varphi =4\pi G_N (\rho -\overline{\rho }), \end{aligned}$$

$\triangle =\delta ^{\alpha \beta }\partial ^2/(\partial x^{\alpha }\partial x^{\beta })$ stands for the Laplace operator, $G_N$ is the Newtonian gravitational constant, and $\rho$ represents the rest mass density in the comoving coordinates, being time-independent within the adopted accuracy, while $\overline{\rho }$ denotes its constant average value. Here both the nonrelativistic and the weak field limits are applied, which means that peculiar velocities of inhomogeneities (galaxies) are negligibly small in comparison with the speed of light, and the metric corrections are much smaller than the corresponding background metric coefficients (i.e. $|\Phi |\ll 1$). The function $\Phi$ given by (2.2) satisfies the following system of linearized Einstein equations of the scalar perturbations theory (see, e.g., [8, 9]):

2.3 $$\begin{aligned} \triangle \Phi -3{\mathcal H}(\Phi '+{\mathcal H}\Phi )=\frac{4\pi G_N}{c^4} a^2(\delta \varepsilon _{\mathrm {mat}}+\delta \varepsilon _{\mathrm {rad}}), \end{aligned}$$

2.4 $$\begin{aligned} \frac{\partial }{\partial x^{\beta }}(\Phi '+{\mathcal H}\Phi )=0, \end{aligned}$$

2.5 $$\begin{aligned} \Phi ''+3{\mathcal H}\Phi '+(2{\mathcal H}'+{\mathcal H}^2)\Phi =\frac{4\pi G_N}{c^4} a^2\delta p_{\mathrm {rad}}. \end{aligned}$$

Here the prime denotes the derivative with respect to the conformal time $\eta$, $\mathcal {H}=a'/a$, $\delta \varepsilon _{\mathrm {rad}}=-3\overline{\rho }\varphi /a^4$, and $\delta p_{\mathrm {rad}}=\delta \varepsilon _{\mathrm {rad}}/3$ represent the perturbations of the energy density and pressure of radiation (the corresponding unperturbed/average quantities are neglected) while $\delta \varepsilon _{\mathrm {mat}}=(\rho -\overline{\rho })c^2/a^3+3\overline{\rho }\varphi /a^4$ is the perturbation of the energy density of the completely nonrelativistic matter (the corresponding unperturbed/average quantity reads $\overline{\varepsilon }_{\mathrm {mat}}=\overline{\rho }c^2/a^3$).

The enumerated results accord with [10, 11] as well as [12, 13]. In addition to them, let us mention the fact that the equations expressing the energy conservation (see, e.g., [9]) hold true with the adopted accuracy for both the nonrelativistic matter and the radiation:

2.6 $$\begin{aligned} \delta \varepsilon '_{\mathrm {mat}}+3\mathcal {H}\delta \varepsilon _{\mathrm {mat}}-3\overline{\varepsilon }_{\mathrm {mat}}\Phi '=0, \end{aligned}$$

2.7 $$\begin{aligned} \delta \varepsilon '_{\mathrm {rad}}+3\mathcal {H}(\delta \varepsilon _{\mathrm {rad}}+\delta p_{\mathrm {rad}})=0, \end{aligned}$$

as one can easily verify by the proper direct substitutions.

In [11] the solution of the Poisson equation (2.2) for the gravitational potential $\varphi$ (in the comoving coordinates) is presented in the standard mathematical physics manner as follows:

2.8 $$\begin{aligned} \varphi =-G_N\int _{V'} \frac{\rho (\mathbf{r}')-\overline{\rho }}{|\mathbf{r}-\mathbf{r}'|}\mathrm{d}V'. \end{aligned}$$

Below we focus attention on the extremely important problematic aspect of this commonly used presentation.

## Infinite- and finite-range gravitational potentials

**Fig. 1:**
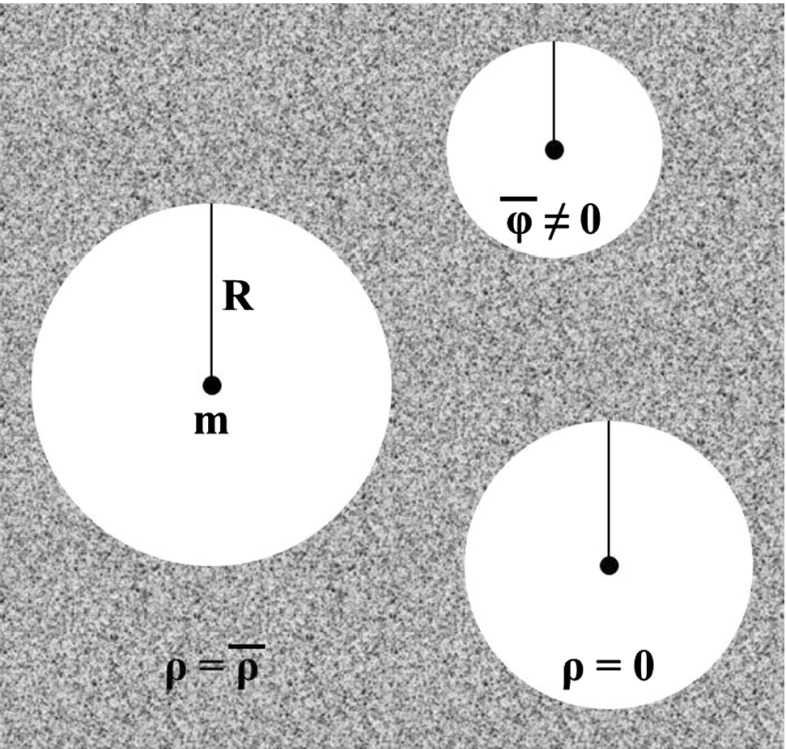
The mass distribution characterized by the nonzero average value of the infinite-range gravitational potential

In the case of the infinite-range gravitational potential (2.8) there is a simple example of the mass distribution leading to nonzero average values of cosmological perturbations. It bears a direct relation to Einstein–Straus/Swiss-cheese models (see, e.g., the recent papers [14, 15]). This artificial, but instructive distribution (described also in [1, 2]) is shown in Fig. 1. The Universe is supposed to be filled with an infinite number of empty spheres ($\rho =0$) with the exception of point-like masses in their centers, embedded in the homogeneous background ($\rho =\overline{\rho }$). The radius *R* of a given sphere is interconnected with the mass *m* in its center: $m=4\pi \overline{\rho }R^3/3$ (all matter from each sphere is concentrated in its center, so the average rest mass density of such Universe remains equal to $\overline{\rho }$, as it certainly should be).

In order to determine the gravitational potential $\varphi$ corresponding to the given sphere, one can solve the Poisson equation (2.2) with the appropriate boundary conditions $\varphi (R)=0$, $\mathrm{d}\varphi /\mathrm{d}r(R)=0$ or use the standard prescription (2.8). The result is the same: inside the sphere (the region I)

3.1 $$\begin{aligned} \varphi _{\mathrm {I}}=2\pi G_N\overline{\rho }\left( R^2-\frac{r^2}{3}\right) -\frac{G_Nm}{r}, \end{aligned}$$

while outside the sphere (the region II)

3.2 $$\begin{aligned} \varphi _{\mathrm {II}}=\frac{4}{3}\pi \overline{\rho }R^3\frac{G_N}{r}-\frac{G_Nm}{r}=0. \end{aligned}$$

Averaging the derived function (3.1) over the volume $V=4\pi R^3/3$ of the sphere, we immediately obtain the senseless result

3.3 $$\begin{aligned} \overline{\varphi }_{\mathrm {I}}=\frac{1}{V}\int _V \varphi _{\mathrm {I}} \mathrm{d}V=-\frac{3G_Nm}{10R}\ne 0, \end{aligned}$$

meaning that the standard prescription (2.8) can lead to unreasonable nonzero average values of cosmological perturbations. One can naively suppose that the result (3.3) is true only for the considered region of the finite volume *V*, while averaging over the infinite volume saves the situation. This argumentation is apparently wrong since there is an infinite number of such regions in the model under consideration, and each of them makes a nonzero (negative) contribution when averaging over the infinite volume. Thus, the average value will be again nonzero (negative).

Trying to save the situation in a different way, one can also change the boundary condition $\varphi (R)=0$ for the Poisson equation (2.2). Namely, one can require that $\varphi (R)=\mathrm {const}\ne 0$ instead, and then adjust this constant in order to satisfy the desired condition $\overline{\varphi }=0$ when averaging over the infinite volume containing an infinite number of regions depicted in Fig. 1. Actually, such a change is equivalent to simply adding this constant in the right-hand side of (2.8). Again, these arguments are apparently wrong. The zero boundary value $\varphi (R)=0$ is in concordance with the standard formula (2.8), which, in its turn, agrees with the generally accepted mathematical physics requirement that any fluctuation $\delta \rho$ produces a decreasing gravitational potential vanishing at spatial infinity. In this connection, if one changes the boundary value or, equivalently, adds some nonzero constant in the right-hand side of (2.8), then such an additional term has unclear physical interpretation and no evident source. It breaks the superposition principle and certainly contradicts a natural demand of vanishing cosmological perturbations in the absence of inhomogeneities (i.e. when $\delta \rho =0$).

Of course, since the function $\Phi$ describes the deviation of the metric coefficients in (2.1) from the corresponding average quantities, its own average value must be equal to zero: $\overline{\Phi }=0$. The same statement must hold true for $\varphi$, $\delta \varepsilon _{\mathrm {rad}}$, etc. The discovered indubitable disadvantage of the formula (2.8) should not be ignored in the modern *N*-body simulations (along with [11, 12]; see [16]).

It is important to remark that the nonzero time-dependent contribution $\overline{\Phi }$ in the averaged metric

$$\begin{aligned} a^2[(1+2\overline{\Phi })\mathrm{d}\eta ^2-(1-2\overline{\Phi })\delta _{\alpha \beta }\mathrm{d}x^{\alpha }\mathrm{d}x^{\beta }] \end{aligned}$$

cannot be eliminated by a coordinate transformation $\eta \rightarrow \eta +\epsilon (\eta )$, $x^{\alpha }\rightarrow x^{\alpha }(1-\lambda )$, where $\epsilon (\eta )$ is some function of the first order of smallness and $\lambda$ is some constant of the same order [17–19]. Really, if such a possibility of elimination had existed, then, according to [18], the following equations would have been true: $\overline{\Phi }=\epsilon '+\mathcal {H}\epsilon$ and $\overline{\Phi }=\lambda -\mathcal {H}\epsilon$. However, taking into account that, as follows directly from (2.2), $\overline{\Phi }\sim 1/a$, one can easily prove that these equations are consistent only in the case $\mathcal {H}'=\mathcal {H}^2$, and this equality does not hold true in the Universe, which is supposed to be filled not only with the dark energy in the form of the $\Lambda$-term, but also with the nonrelativistic matter. The latter, as is generally known, makes different contributions in the background Friedmann equations for $\mathcal {H}^2$ and $\mathcal {H}'$. Thus, the considered coordinate transformation does not help either.

In order to avoid this difficulty, let us introduce the finite-range gravitational potential by modifying (2.8) as follows:

3.4 $$\begin{aligned} \varphi =-G_N\int _{V'} \frac{\rho (\mathbf{r}')-\overline{\rho }}{|\mathbf{r}-\mathbf{r}'|}\Theta (R_*-|\mathbf{r}-\mathbf{r}'|)\mathrm{d}V', \end{aligned}$$

where $\Theta$ represents the Heaviside step function, and $R_*$ is some positive (generally speaking, time-dependent) cutoff distance which may be associated with the particle horizon. This modification is inspired by a similar cutoff when describing propagation of electro-magnetic or gravitational waves: the field is nonzero only in those points which have received the corresponding signal, even if its source is resting. Then the taken step may be interpreted as making the Newtonian classical mechanics more precise by supplementing it with the special (not general!) relativity idea of the signal propagation speed finiteness. Here peculiar velocities of inhomogeneities (galaxies) are completely neglected as before, and the first-order perturbation theory holds true (the gravitational field described by the metric corrections remains weak). Evidently, the introduction of the formula (3.4) instead of the predecessor without the Heaviside step function represents a particular modification of the gravitation theory where scalar modes are no longer instantaneous.

Applying (3.4) to the mass distribution under consideration and restricting ourselves to the case $R_*>2R$, we get (3.1) and (3.2) for the gravitational potentials $\varphi _{\mathrm {I}}$ and $\varphi _{\mathrm {II}}$ in the regions I ($0<r<R$) and II ($R<r<R_*-R$), respectively, as well as

3.5 $$\begin{aligned} \varphi _{\mathrm {III}}=\frac{\pi G_N\overline{\rho }}{r}\left[ R^2(R_*-r)-\frac{2}{3}R^3-\frac{1}{3}(R_*-r)^3\right] \end{aligned}$$

in the region III ($R_*-R<r<R_*$), while

3.6 $$\begin{aligned} \varphi _{\mathrm {IV}}=\frac{\pi G_N\overline{\rho }}{r}\left[ R^2(R_*-r)+\frac{2}{3}R^3-\frac{1}{3}(R_*-r)^3\right] \end{aligned}$$

in the region IV ($R_*<r<R_*+R$). Finally,

3.7 $$\begin{aligned} \varphi _{\mathrm {V}}=0 \end{aligned}$$

in the region V ($r>R_*+R$).

Now the direct calculation gives the desired result:

3.8 $$\begin{aligned}&\int \limits _0^{R}\varphi _{\mathrm {I}}(r)r^2\mathrm{d}r+\int \limits _R^{R_*-R}\varphi _{\mathrm {II}}(r)r^2\mathrm{d}r\nonumber \\&\quad +\int \limits _{R_*-R}^{R_*}\varphi _{\mathrm {III}}(r)r^2\mathrm{d}r+\int \limits _{R_*}^{R_*+R}\varphi _{\mathrm {IV}}(r)r^2\mathrm{d}r=0, \end{aligned}$$

therefore, $\overline{\varphi }=0$, as it certainly should be. Thus, the use of the finite-range gravitational potential (3.4) instead of the infinite-range one (2.8) leads to reasonable zero average values of cosmological perturbations. This advantage of the proposed formula (3.4) in comparison with (2.8) may be taken into account when simulating the behavior of *N*-body systems.

Let us mention that in the framework of the extension of the $\Lambda$CDM model, assuming the presence in the Universe of the additional constituent (namely, quintessence) with the linear equation of state $\varepsilon _q=\omega _qp_q$ with the constant parameter $\omega _q=-1/3$, the discussed problem of nonzero average values of cosmological perturbations in the case of the infinite-range gravitational potential is resolved in a different manner: quintessence fluctuations around a point-like nonrelativistic matter inhomogeneity cause the Yukawa form of its potential instead of the Newtonian one, and the average value of the total potential produced by all inhomogeneities is really zero [20], irrespective of the interaction range and its cutoff.

The other extension, assuming a negative spatial curvature, is also characterized by the potential of a point-like inhomogeneity, similar to the Yukawa one, so the average value of the total potential is again zero [2].

Returning to the conventional model under consideration and introducing the dimensionless quantities $\chi =r/R$, $\xi =R_*/R$, and $\widetilde{\varphi }=\varphi /(4 \pi G_N\overline{\rho }R^2/3)$, from (3.1), (3.5) and (3.6) we obtain, respectively,

3.9 $$\begin{aligned} \widetilde{\varphi }_{\mathrm {I}}(\chi )=\frac{3}{2}-\frac{\chi ^2}{2}-\frac{1}{\chi }, \end{aligned}$$

3.10 $$\begin{aligned} \widetilde{\varphi }_{\mathrm {III}}(\chi )=\frac{3(\xi -\chi )}{4\chi }-\frac{1}{2\chi }-\frac{(\xi -\chi )^3}{4\chi }, \end{aligned}$$

3.11 $$\begin{aligned} \widetilde{\varphi }_{\mathrm {IV}}(\chi )=\frac{3(\xi -\chi )}{4\chi }+\frac{1}{2\chi }-\frac{(\xi -\chi )^3}{4\chi }, \end{aligned}$$

while from (3.2) and (3.7) it trivially follows that $\widetilde{\varphi }_{\mathrm {II}}=\widetilde{\varphi }_{\mathrm {V}}=0$. The spherical surfaces $r=R;\, R_*-R;\, R_*+R$ (where the function $\varphi (r)$ is smooth, as one can easily demonstrate) correspond to $\chi =1;\, \xi -1;\, \xi +1$ (where the smoothness conditions are satisfied for the function $\widetilde{\varphi }(\chi )$). At the same time on the surface $r=R_*$ corresponding to $\chi =\xi$ and delimiting the spatial regions III and IV there is a foreseeable jump discontinuity:

3.12 $$\begin{aligned} \widetilde{\varphi }_{\mathrm {III}}(\xi )-\widetilde{\varphi }_{\mathrm {IV}}(\xi )=-\frac{1}{\xi },\quad \frac{\mathrm{d}\widetilde{\varphi }_{\mathrm {III}}}{\mathrm{d}\chi }(\xi )-\frac{\mathrm{d}\widetilde{\varphi }_{\mathrm {IV}}}{\mathrm{d}\chi }(\xi )=\frac{1}{\xi ^2}.\nonumber \\ \end{aligned}$$

Really, on the surface under consideration the action of the central mass gravitational field ends, so the result (3.12) is expected, and it does not relate to the modification (3.4) itself. In terms of the function $\varphi (r)$ and its derivative $\mathrm{d}\varphi /\mathrm{d}r$ this result can be trivially rewritten as follows:

3.13 $$\begin{aligned}&{\varphi }_{\mathrm {III}}(R_*)-{\varphi }_{\mathrm {IV}}(R_*)=-\frac{G_Nm}{R_*},\nonumber \\&\frac{\mathrm{d}{\varphi }_{\mathrm {III}}}{\mathrm{d}r}(R_*)-\frac{\mathrm{d}{\varphi }_{\mathrm {IV}}}{\mathrm{d}r}(R_*)=\frac{G_Nm}{R_*^2}. \end{aligned}$$

**Fig. 2:**
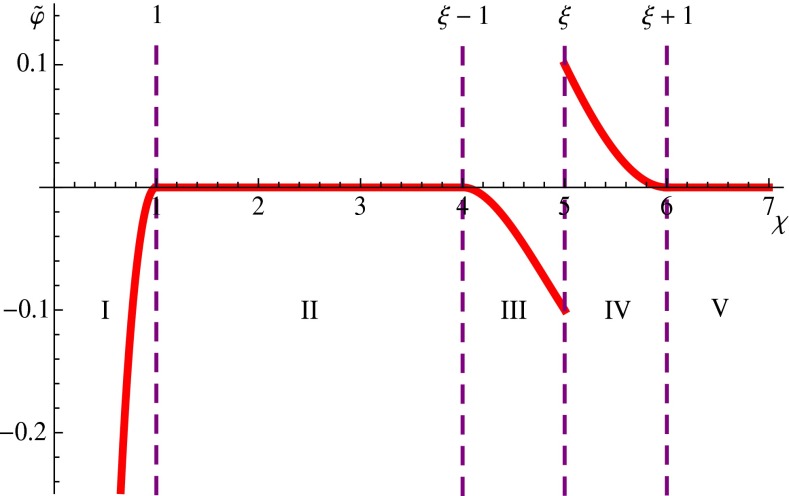
The finite-range gravitational potential as a function of the radial distance

The dependence $\widetilde{\varphi }(\chi )$ is depicted in Fig. 2 (for solely illustrative purposes the choice $\xi =5$ is made).

## Conclusion

We have proven that for some matter distributions at the late stage of the Universe evolution (see, e.g., Fig. 1) the nonrelativistic gravitational potential can be characterized by the nonzero average value if the standard formula (2.8) is applied. This situation is absolutely inadmissible, so in order to resolve this challenge we have cut off the gravitational potential and with the help of the modified formula (3.4) obtained the desired result $\overline{\varphi }=0$. Evidently, it is valid not only for the considered concrete density profile but also for an arbitrary one. It is important to stress that our conclusion is correct provided that the made assumptions concerning the FLRW geometry as an average one and the comoving volume averaging as an appropriate averaging procedure are valid. Then the suggested application of the finite-range potentials instead of the infinite-range ones can improve the quality and precision of the cosmological simulations with respect to their representation of the physical reality.
